# Epidemiological profile of congenital hypothyroidism at a southern Brazilian state

**DOI:** 10.20945/2359-3997000000606

**Published:** 2023-05-10

**Authors:** Márcia Inês Boff, Cristiane Kopacek, Vandrea Carla de Souza, Sabliny Carreiro Ribeiro, Edmundo Kreisner, Paula Regla Vargas, Livia Silveira Mastella, José Mauro Madi, Simone Martins de Castro, Rosa Maria Rahmi

**Affiliations:** 1 Universidade de Caxias do Sul Caxias do Sul RS Brasil Universidade de Caxias do Sul, Caxias do Sul, RS, Brasil; 2 Hospital Materno-Infantil Presidente Vargas Triagem Neonatal do Estado do Rio Grande do Sul Porto Alegre RS Brasil Serviço de Referência em Triagem Neonatal do Estado do Rio Grande do Sul, Hospital Materno-Infantil Presidente Vargas, Porto Alegre, RS, Brasil; 3 Universidade Federal de Ciências da Saúde de Porto Alegre Programa de Pós-graduação em Pediatria Porto Alegre RS Brasil Programa de Pós-graduação em Pediatria, Universidade Federal de Ciências da Saúde de Porto Alegre, Porto Alegre, RS, Brasil; 4 Universidade Federal do Rio Grande do Sul Porto Alegre RS Brasil Universidade Federal do Rio Grande do Sul, Porto Alegre, RS, Brasil; 5 Universidade de Caxias do Sul Departamento de Pediatria Caxias do Sul RS Brasil Departamento de Pediatria, Universidade de Caxias do Sul, Caxias do Sul, RS, Brasil; 6 Secretaria Municipal de Saúde de Porto Alegre Porto Alegre RS Brasil Secretaria Municipal de Saúde de Porto Alegre, Porto Alegre, RS, Brasil; 7 Universidade de Caxias do Sul Hospital Geral de Caxias do Sul Departamento de Obstetrícia e Ginecologia Caxias do Sul RS Brasil Departamento de Obstetrícia e Ginecologia, Hospital Geral de Caxias do Sul, Universidade de Caxias do Sul, Caxias do Sul, RS, Brasil; 8 Universidade de Caxias do Sul Departamento de Endocrinologia Caxias do Sul RS Brasil Departamento de Endocrinologia, Universidade de Caxias do Sul, Caxias do Sul, RS, Brasil

**Keywords:** Congenital hypothyroidism, neonatal screening, public health, incidence

## Abstract

**Objective:**

To determine the incidence of congenital hypothyroidism (CH) over a 10-year period at the Reference Service in Neonatal Screening of the state of Rio Grande do Sul (RSNS-RS).

**Subjects and methods:**

Historical cohort study including all newborns screened for CH by the RSNS-RS from January 2008 until December 2017. Data of all newborns with neonatal TSH (neoTSH; heel prick test) values ≥ 9 mIU/L were collected. According to neoTSH values, the newborns were allocated into two groups: Group 1 (G1), comprising newborns with neoTSH ≥ 9 mIU/L and serum TSH (sTSH) < 10 mIU/L, and Group 2 (G2), comprising those with neoTSH ≥ 9 mIU/L and sTSH ≥ 10 mIU/L.

**Results:**

Of 1,043,565 newborns screened, 829 (0.08%) had neoTSH values ≥ 9 mIU/L. Of these, 284 (39.3%) had sTSH values < 10 mIU/L and were allocated to the G1 group, while 439 (60.7%) had sTSH ≥ 10 mIU/L and were allocated to the G2 group, and 106 (12.7%) were considered missing data. The overall incidence of CH was 42.1 per 100,000 newborns screened (95% confidence interval [CI] 38.5-45.7/100,000) or 1:2377 screened newborns. The sensibility and specificity of neoTSH ≥ 9 mIU/L were 97% and 11%; of neoTSH 12.6 mUI/L, 73% and 85% respectively.

**Conclusion:**

In this population, the incidence of permanent and transitory CH was 1:2377 screened newborns. The neoTSH cutoff value adopted during the study period showed excellent sensibility, which matters for a screening test.

## INTRODUCTION

Congenital hypothyroidism (CH), the most frequent congenital endocrine disorder, has devastating effects on brain development if early treatment is not implemented. Primary CH may occur due to thyroid dysgenesis (which includes a spectrum of thyroid developmental abnormalities) or dyshormonogenesis ( *i.e.* , a molecular defect involving thyroid hormonogenesis in a structurally intact gland) ( 1 ).

Neonatal screening programs (NSPs) offer the best strategy for early diagnosis and management of CH and have almost eliminated the occurrence of severe neurodevelopmental deficit associated with a late diagnosis of the disease. The main objective of NSPs is to identify newborns with primary CH. Since the establishment of the CH screening program in the 1970s, neonatal screening has been routinely implemented in developed countries and in some developing countries and has been an enormous public health success ( 2 ). In 2001, the Brazilian Ministry of Health developed the National Neonatal Screening Program (NNSP) in partnership with health departments at each state (and Federal District) and city. The NNSP recommends CH screening by neonatal TSH (neoTSH) measurement (heel prick test), ideally collected between the third and fifth days of life. Neonates with neoTSH results ≥ 10 mIU/L must undergo immediate measurement of serum TSH (sTSH), total T4 (tT4), and free T4 (fT4) obtained from a venous blood sample ( 3 ).

In 2001, the state of Rio Grande do Sul (RS) implemented the Reference Service in Neonatal Screening (RSNS-RS) at the President Vargas Maternal-Infantile Hospital (PVMIH) in the city of Porto Alegre. The RSNS-RS is responsible for local public neonatal screening, covering almost 75% of all statewide live births ( 4 ). Between 2005 and 2017, the service had adopted a neoTSH cutoff value of 9 mIU/L ( 5 ), but since 2018, the cutoff value has been lowered to 6 mIU/L.

The decreased neoTSH cutoff value has resulted in a higher incidence of CH, mostly due to the identification of children with neonatal hypothyroidism who would otherwise remain undetected and progress to mild permanent thyroid dysfunction ( 6 ). Other factors have also been attributed to the increased CH incidence, such as changes in screening protocols, planned repetition of screening in premature newborns, changes in demographic patterns, and increased frequency of multiple pregnancy ( 7 – 10 ). Still, further studies are needed to examine this phenomenon of increased CH incidence.

The global incidence of CH was about 1:6000 live births before the implementation of CH screening programs ( 11 ). During the first decade after implementation of the programs, the incidence increased to about 1:3800-1:4000 live births ( 2 ), and recent data have shown an even further increase to 1:1600-1:2800 ( 9 , 12 , 13 ).

In 2015, the NNSP of the Brazilian Ministry of Health reported a national incidence of CH of about 1:2595-1:4795 live births ( 3 ). In 2009, Magalhães and cols. reported a CH incidence of 1:2595 in a population of 197,265 children screened by the NSP of the Ribeirão Preto Medical School Hospital (affiliated with the University of São Paulo) adopting a neoTSH cutoff value of 10 μIU/mL ( 14 ). Using a cutoff value of 5.0 μIU/mL, Silvestrin and cols. reported a prevalence of CH of 1:2234 live births in a population screened by the RSNS of the state of Mato Grosso ( 15 ).

Given the importance of local data in improving public health management, periodic evaluation of protocols should be carried out, and the results should be reported. To contribute with epidemiological data for the state of RS, the aim of the present study was to determine the incidence of CH cases in the population of newborns screened over a 10-year period in which the RSNS-RS used a neoTSH cutoff value of 9 mIU/L. The study also aimed at analyzing the accuracy of the neoTSH cutoff value in this population.

## SUBJECTS AND METHODS

### Design and population

This historical cohort study was conducted with data collected from all newborns screened for CH by the RSNS-RS from January 2008 through December 2017. The protocol of the local NSP includes measurement of neoTSH from dried blood samples collected on filter paper from all newborns in the neonatal period. NeoTSH levels were measured in blood spots dried on filter paper (S&S 903) using fluorescence immunoassay (AutoDelfia Neonatal TSH) and GSP Neonatal TSH (PerkinElmer, Turku, Finland) kits. The linearity range of neoTSH was 0.66 to 375 mIU/L. In the study period, the cutoff value adopted for neoTSH was 9 mIU/L, and all newborns with neoTSH levels ≥ 9 mIU/L were referred for clinical evaluation and sTSH measurement. Newborns with sTSH levels ≥ 10 mIU/L were referred for evaluation by a medical team specialized in CH management.

All newborns with neoTSH ≥ 9 mIU/L were considered to be at risk for CH and had their data collected. Data from newborns with neoTSH < 9 mIU/L or neoTSH ≥ 9 mIU/L without record of sTSH values were excluded. Since data from newborns with neoTSH values < 9 mIU/L were not recorded, these data were not collected. The data were allocated into the following two groups: Group 1 (G1), in which values of neoTSH were ≥ 9 mIU/L and sTSH were < 10 mIU/L and Group 2 (G2), in which values of neoTSH were ≥ 9 mIU/L and sTSH were ≥ 10 mIU/L.

The following maternal variables were collected: age at the beginning of pregnancy, type of delivery, occurrence of multiple pregnancy, gestational complications (including the development of thyroid disease during pregnancy) and other complications (use of Lugol's iodine solution, HIV infection, diabetes, preeclampsia, eclampsia, smoking and drug abuse, anemia, and urinary infection), diagnosis of thyroid disease before pregnancy, and parental consanguinity. Newborn variables collected included the city of origin, sex, gestational age (>37, 34-36, and <34 weeks), birth weight (in grams), family history of thyroid disease (father and/or second-degree family member), occurrence of congenital malformation and presence of genetic syndrome (physical examination and/or proper complementary exams were used for malformations evaluation).

The protocol of the study was approved by the Research Ethics Committee at PVMIH [(CAAE 80323917.6.0000.5329) (Porto Alegre, Brazil)]. The study was conducted according to guidelines and norms regulating human research.

### Statistical analysis

Continuous variables were expressed as median and interquartile range. Histograms and the Kolmogorov-Smirnov and Shapiro-Wilk tests were used to test quantitative data for normality of distribution. Equality of variances in both groups (G1 and G2) was tested using Levene's test. Qualitative variables were compared using the chi-square test or Fisher's exact test, while median values were compared using the Wilcoxon test. The area under the receiver operating characteristic (ROC) curves (AUC) was used to determine the ability of neoTSH to discriminate patients with sTSH values ≥ 10 mIU/L from those with values < 10 mIU/L. Logistic regression was performed to estimate the odds ratio (OR) and 95% confidence intervals (95% CI) of associations between explanatory variables and TSH values ≥ 10 mIU/L. A stepwise method was used to select explanatory variables with *p* values below 0.2 (likelihood ratio test). The goodness-of-fit of the model was verified using the Hosmer and Lemeshow test. *P* values below 0.05 were considered significant. The statistical analyses were performed using R for Windows, version 4.0.2 (R-Cran project, http://cran.r-project.org/ ).

## RESULTS

**Figure 1:**
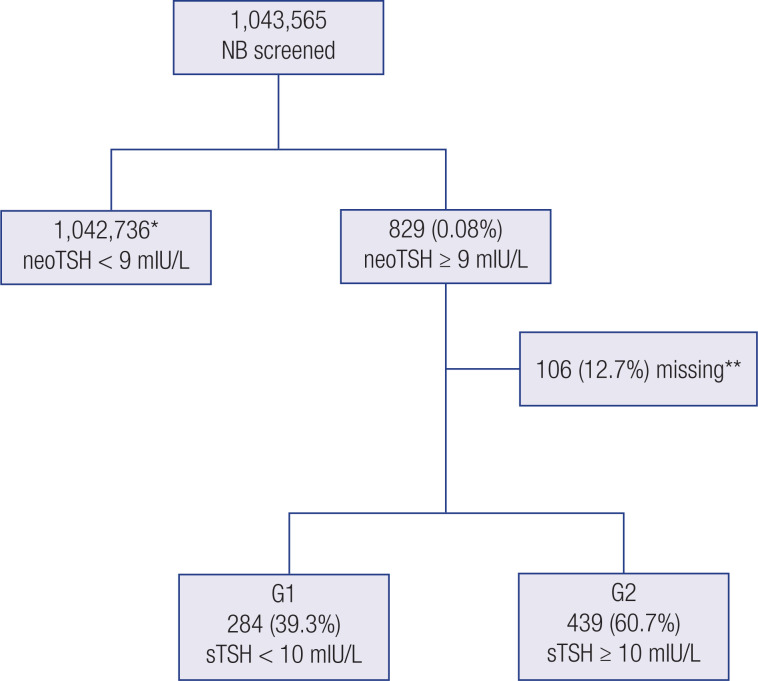
Flowchart outlining the inclusion of the study sample. *Newborns recruited for sTSH measurement and clinical evaluation. **Missing data due to the absence of sTSH records. Abbreviations – NB: newborn; neoTSH: neonatal TSH; sTSH: serum TSH; G1: Group 1; G2: Group 2.

Of 1,395,925 live births registered in RS between January 1, 2008, and December 31, 2017 ( 4 ), 1,043,565 (74.7%) were screened by the RSNS-RS program. A total of 829 (0.08%) newborns had neoTSH values ≥ 9 mIU/L and were referred for sTSH measurement. Of these, 284 (39.3%) had sTSH levels < 10 mIU/L and were allocated to the G1 group, while 439 (60.7%) had sTSH levels ≥ 10 mIU/L and were allocated to the G2 group. In all, 106 (12.7%) newborns had no sTSH results in their medical records and were excluded from the analysis. Newborns with neoTSH values < 9 mIU/L (n = 1,042,736) were discharged ( Figure 1 ). The median time between birth and collection of samples for neoTSH measurement was 6 days (interquartile interval 4-8 days). The percentage of newborns that was screened between 3 and 5 days of life was 46.7%. The median newborn's age at the first consultation was 21 days (interquartile interval 18-26 days).

**Figure 2:**
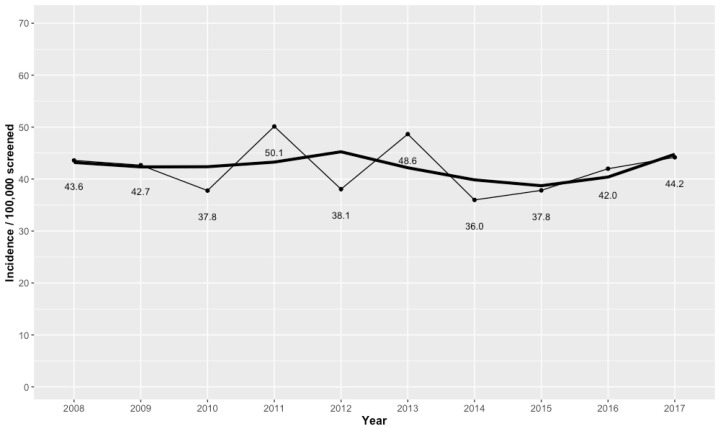
Trend graph outlining the overall yearly incidence rates of congenital hypothyroidism at the RSNS-RS program in 2008-2017. The thin line represents the incidence per 100,000 screened newborns, and the thick line represents the smoothed trend.

The overall incidence of CH was 42.1 per 100,000 screened newborns (95% CI 38.5-45.7/100,000) or 1:2377 newborns screened in the study period. The yearly incidences of CH per 100,000 screened newborns were 43.6 in 2008, 42.7 in 2009, 37.8 in 2010, 50.1 in 2011, 38.1 in 2012, 48.6 in 2013, 36.0 in 2014, 37.8 in 2015, 42.0 in 2016, and 44.2 in 2017 ( Figure 2 ).

Table 1 shows the results of univariate analysis considering the maternal characteristics (maternal age, cesarean delivery, twin pregnancy, gestational complications, thyroid disease, and parental consanguinity) in both G1 and G2, showing no significant differences between groups.

**Table 1:** Univariate analysis of the maternal characteristics in Groups 1 and 2

Variables		G1 (n = 284) n (%)	G2 (n =4 39) n (%)	OR (95% CI)	*P* value
Maternal age (years)					
	<35	209 (73.6)	354 (80.6)		
	≥35	44 (15.5)	57 (13.0)	0.76 (0.49-1.17)	0.22
	MD d	31 (10.9)	28 (6.4)		
Cesarean delivery					
	No	100 (35.2)	149 (34.0)		
	Yes	123 (43.3)	213 (48.5)	1.16 (0.82-1.62)	0.38
	MD d	61 (21.5)	77 (17.5)		
Twin pregnancy					
	No	264 (92.9)	414 (94.3)		
	Yes	5 (1.8)	10 (2.3)	1.27 (0.44-4.13)	0.66
	MD d	15 (5.3)	15 (3.4)		
Gestational complications a					
	No	149 (52.5)	269 (61.3)		
	Yes	106 (37.3)	144 (32.8)	0.75 (0.54-1.03)	0.08
	MD d	29 (10.2)	26 (5.9)		
TD in pregnancy b					
	No	88 (31.0)	120 (27.4)		
	Yes	10 (3.5)	19 (4.3)	1.39 (0.62-3.25)	0.42
	MD d	186 (65.5)	300 (68.3)		
Prior TD c					
	No	232 (81.7)	383 (87.2)		
	Yes	19 (6.7)	31 (7.1)	0.98 (0.55-1.81)	0.96
	MD d	33 (11.6)	25 (5.7)		
Parental consanguinity					
	No	238 (83.8)	390 (88.8)		
	Yes	9 (3.2)	14 (3.2)	0.94 (0.4-2.31)	0.9
MD d		37 (13.0)	35 (8.0)		

Univariate logistic regression model. G1: group of newborns with sTSH < 10 mIU/L. G2: group of newborns with sTSHs ≥ 10 mIU/L. OR: odds ratio; 95% CI: 95% confidence interval.

a Use of Lugol's iodine solution, HIV infection, gestational diabetes, preeclampsia, eclampsia, smoking, use of illicit drugs, anemia, urinary tract infection.

b TD in pregnancy: Thyroid disease diagnosed during pregnancy.

c Prior TD: Thyroid disease diagnosed before pregnancy.

d MD: Missing data of each variable.

Table 2 shows the results of univariate analysis considering neonatal variables in both G1 and G2. Male newborns had a 46% lower probability of having a sTSH level ≥ 10 mIU/L (OR 0.54, 95% CI 0.39-0.73, *p* < 0.001) compared with female newborns. The occurrence of sTHS levels ≥ 10 mIU/L was lower in newborns with gestational age 34-36 weeks (late prematurity) compared with ≥ 37 weeks (OR 0.53, 95% CI 0.31-0.9, *p* = 0.01). No significant differences were observed between groups regarding rates of gestational age < 34 weeks, birth weight, thyroid disease in family members, congenital anomalies, or genetic syndrome.

**Table 2:** Univariate analysis of the neonatal characteristics in Groups 1 and 2

Variables		G1 (n = 284) n (%)	G2 (n = 439) n (%)	OR	*P* value
Male sex					
	No	120 (42.3)	251 (57.2)		
	Yes	159 (55.9)	180 (41.0)	0.54 (0.39-0.73)	<0.001
	MD c	5 (1.8)	8 (1.8)		
	Weight (g)	3117 a	3197 a	1.0 (0.99-1.00)	0.7
Gestational age (weeks)					
	≥37	191 (67.3)	325 (74.0)		
	34-36+6	34 (11.9)	31 (7.1)	0.53 (0.31-0.9)	0.01
	<34	6 (2.1)	9 (2.0)	0.88 (0.31-2.66)	0.81
	MD c	53 (18.7)	74 (16.9)		
TD in family member b					
	No	186 (65.5)	306 (69.7)		
	Yes	61 (21.5)	107 (24.4)	1.04 (0.73-1.49)	0.80
	MD c	37 (13.0)	26 (5.9)		
Congenital anomalies					
	No	244 (85.9)	403 (91.8)		
	Yes	11 (3.9)	16 (3.7)	0.88 (0.4-1.98)	0.75
	MD c	29 (10.2)	20 (4.5)		
Genetic syndrome					
	No	241 (84.9)	395 (90.0)		
	Yes	12 (4.2)	21 (4.8)	1.06 (0.52-2.2)	0.86
MD c		31 (10.9)	23 (5.2)		

Univariate logistic regression model. G1: Group 1, comprising newborns with sTSH values < 10 mIU/L. G2: Group 2, comprising newborns with sTSH values ≥ 10 mIU/L. OR: odds ratio; 95% CI: 95% confidence interval. (g): grams.

a Median.

b TD in family member: Thyroid disease in first- and second-degree family members.

c MD: Missing data of each variable.

Table 3 shows the results of multivariate logistic regression analysis for variables associated with sTSH levels ≥ 10 mIU/L (CH cases). Male sex (OR 0.52, 95% CI 0.37-0.74, *p* < 0.001) and gestational age between 34 and 36 weeks (OR 0.54, 95% CI 0.31–0.94, *p* = 0.03) were independently associated with fewer occurrences of sTSH values ≥ 10 mIU/L.

**Table 3:** Multivariate logistic regression analysis including variables associated with serum TSH ≥ 10 mIU/L

Variable		OR	95% CI	*P* value
Male sex		0.52	0.37-0.74	<0.001
Gestational age (weeks)				
	≥37 *			
	34-37	0.54	0.31-0.94	0.03
	≤34	0.86	0.26-3.02	0.81
Congenital anomalies				
	No *			
	Yes	0.74	0.28-1.95	0.53
Gestational complications a				
	No *	0.82	0.57-1.17	
	Yes			0.28
Maternal age (years)			0.41-1.08	
	<35 *			
	≥35	0.67		0.10

* Reference category.

a Use of Lugol's iodine solution, HIV infection, gestational diabetes mellitus, preeclampsia, eclampsia, smoking, use of illicit drugs, anemia, urinary tract infection. OR: odds ratio; 95% CI: 95% confidence interval.

**Figure 3:**
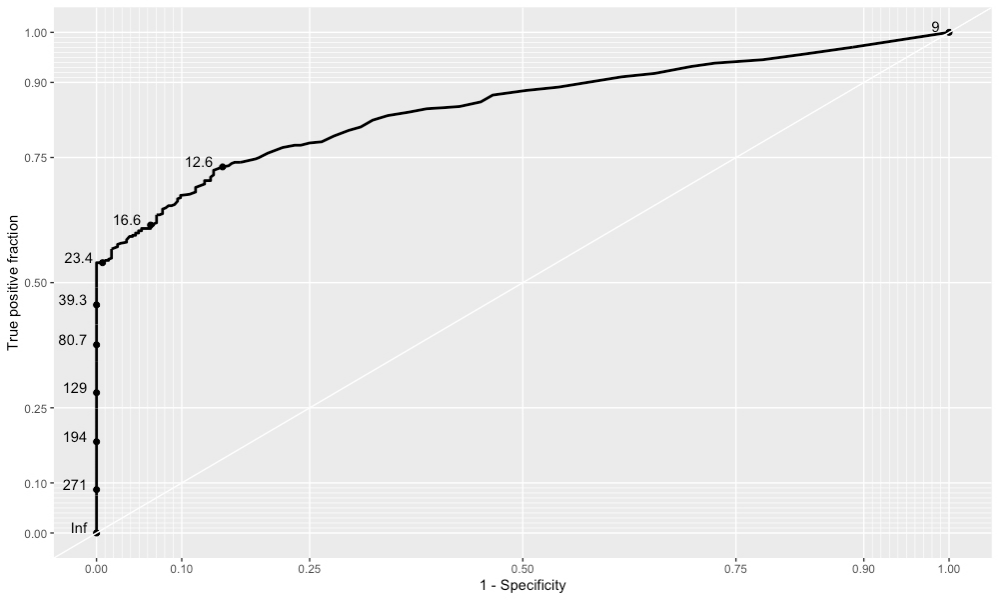
Receiver operating characteristic curve (ROC) and area under curve (AUC) of neonatal TSH values.

The ROC curve ( Figure 3 ) shows the ability of neoTSH in identifying newborns with sTSH values < 10 mIU/L (G1) and ≥ 10 mIU/L (G2). The best cutoff value for neoTSH was 12.6 mIU/L in the entire study cohort, and the AUC was 0.85 (95% CI 0.8231-0.8776).

Table 4 shows the sensitivity, specificity, positive predictive value (PPV), and negative predictive value (NPV) of different cutoff values for neoTSH in the study cohort.

**Table 4:** Sensitivity, specificity, positive predictive value, and negative predictive value of different neoTSH cutoff values

neoTSH (mIU/L)	Sensitivity (CI * )	Specificity (CI * )	PPV (CI * )	NPV (CI * )
9.05	0.97 (0.95-0.98)	0.11 (0.07-0.15)	–	–
10	0.88 (0.85-0.91)	0.49 (0.43-0.55)	0.73 (0.69-0.76)	0.73 (0.67-0.79)
12	0.73 (0.68-0.77)	0.85 (0.81-0.89)	0.88 (0.85-0.91)	0.67 (0.62-0.72)
15	0.64 (0.60-0.69)	0.91 (0.88-0.95)	0.92 (0.89-0.95)	0.62 (0.58-0.67)
20	0.56 (0.52-0.61)	0.98 (0.96-0.99)	0.98 (0.96-0.99)	0.59 (0.55-0.63)
40	0.45 (0.40-0.49)	1.00 (1.00-1.00)	1.00 (1.00-1.00)	0.54 (0.49-0.58)

* CI: 95% confidence interval; PPV: positive predictive value; NPV: negative predictive value.

## DISCUSSION

The present study, which had a considerable sample size, showed the incidence of CH cases in a southern Brazilian state over a 10-year period from 2008 to 2017. The local incidence of CH cases (transient and permanent) was 42.1 per 100,000 screened cases (1:2377) using a neoTSH cutoff value of 9 mIU/L, indicating good accuracy. Previous data from Kreisner and cols. have shown a CH incidence of 1:2,818 newborns during 2003-2005 in the same NSP as the one in the present study but using a neoTSH cutoff value of 20 mIU/L and a different laboratory methodology ( 16 ). Nascimento and cols. reported that 49 of 148,717 screened newborns were considered to have CH at an NSP in the state of Santa Catarina using a neoTSH cutoff value of 9 mIU/L ( 17 ). Different cutoff values for neoTSH used across the country could account for differences in CH frequencies, as observed in other Brazilian studies. Magalhães and cols. have shown a total incidence of CH of 1:2595 newborns screened in the city of Ribeirão Preto (state of São Paulo) using a neoTSH cutoff value of 10 mIU/L ( 14 ). The NSP of Campinas University (Unicamp) reported a prevalence of CH of 9.13% based on neoTSH values ranging between 5-10 mIU/L ( 18 ). In Sergipe, the total incidence of CH was 1:3,461 ( 19 ), and in the state of Mato Grosso, the incidence was 1:2,234 screened newborns ( 15 ), using neoTSH cutoff values of 5.2 and 5.0 mIU/L, respectively. These findings indicate that the adoption of lower neoTSH cutoff values results in a higher frequency of CH cases. Data from the state of Paraná indicated an increasing prevalence of CH – from 1:4,510 to 1:2,296 newborns – with protocol modifications ( 20 ). In 2020, Nascimento and cols. reported a higher incidence (1:1,560 screened newborns) when a lower neoTSH cutoff value was adopted in the NSP of the state of Santa Catarina ( 21 ).

In Lombardy (Italy), when the neoTSH cutoff value was lowered from 20 mIU/L to 12 mIU/L in 1999 and further decreased to 10 mIU/L in 2002, the incidence of CH increased from 1:2,654 to 1:1,446 newborns ( 6 ). A study conducted over 21 years and involving all Italian neonatal screening centers showed an increase in the CH incidence (transient and permanent) from 1:3000 to 1:1940 newborns concomitant to a decrease in the neoTSH cutoff value from 20 mIU/L to 15 mIU/L ( 13 ). Other studies from Greece ( 22 ) and Quebec ( 12 ) have shown comparable results. Observing the different cut-off points presented nationally and internationally, with a slight increase in incidence, there is a tendency to reduce the cut-off points, however there is still no consensus on the best value of nTSH, and the differences between populations should be observed.

No single cause can explain the increased incidence of CH. McGrath and cols. have shown an increasing incidence of CH in Ireland without any change in neoTSH cutoff value ( 23 ). Similar findings have been reported by Albert and cols. in a study from New Zealand ( 24 ). Olivieri and cols. ( 13 ) have suggested that more than one cause was responsible for the increasing CH incidence in their study, including improvements in neonatal medicine, which have contributed to an increased survival rate of premature newborns. In our study, a gestational age < 34 weeks was not associated with CH; however, we observed that late premature newborns (between 34 and 36 weeks) were less likely to have CH compared with newborns born at 37 weeks or later. Other factors, such as ethnicity and parental consanguinity, have been associated with CH. In the Italian population, the rate of parental consanguinity (first-degree relatives) was significantly higher among newborns with CH of African, Asian, or Hispanic descent compared with Caucasian newborns ( 13 ). Aligned with the results obtained in a study with Iranian newborns ( 25 ), our study also found no significant association between CH cases and parental consanguinity. On the other hand, our study found that the female sex was more frequent among CH cases, as reported in other studies. Medda and cols. ( 26 ) and Knowles and cols. ( 27 ) reported that permanent CH was more common in female than male newborns. Olivieri and cols. reported that the female sex was five times more frequent in CH cases due to dysgenesis but observed no difference between sexes in CH cases due to dyshormonogenesis ( 28 ).

Congenital malformations were not associated with CH cases in our population. However, a previous study by Kreisner and cols. ( 29 ) reported a high prevalence of extrathyroidal malformations in a cohort with permanent primary at the same RSNS as the one in which the present study was conducted, a finding aligned with results reported by Ruíz and cols. ( 20 ) in the state of Paraná and McGrath and cols. ( 23 ) in Ireland. Our study also found no association between CH and maternal age or maternal thyroid disease, aligned with findings reported in an Iranian study ( 25 ) but in opposition to data from a Chinese study ( 30 ).

An important aspect of our results was the good accuracy of the neoTSH cutoff value adopted in the study period, represented by the AUC of 0.85 ( 31 ). However, the best calculated performance was with neoTSH cutoff values ≥ 12.6 mIU/L. The RSNS from Mato Grosso reported an AUC of 0.98 for neoTSH cutoff values ≥ 5 mIU/L ( 15 ). A prospective British study evaluated three neoTSH cutoff values (6 mIU/L, 8 mIU/L, and 10 mIU/L) and found that the best performance was with the value of 8 mIU/L ( 27 ). At the beginning of the neonatal CH screening program in our state, the neoTSH cutoff value used was 20 mIU/L ( 16 ). We observed in our sample that with the adoption of a neoTSH cutoff value of 20 mIU/L, the probability of selecting newborns without CH (sTSH < 10 mIU/L) was close to zero due to the high specificity of this cutoff value (98%; 95% CI 0.96-0.99). These data suggest that treatment could be started as soon as possible for newborns with neoTSH values ≥ 20 mIU/L, even before the results of sTSH become available, as recommended for neoTSH values ≥ 40 mIU/L ( 32 ). Also in our analysis, neoTSH ≥ 40 mIU/L had a PPV of 100%, reinforcing the need for initiation of immediate treatment with results above this value.

Of note, several NSPs have established their neoTSH cutoff values based on the recommendation of collecting blood in filter paper between the third and fifth days of life. However, we observed in our country that the sample collection and analysis take a longer time, hindering proper interpretation of the results ( 18 , 33 ). In our results, less than half of the study population was screened within the recommended lifespan, probably due to poor staff training and public transport, which could be a potential limitation.

An important strength of the present study is the considerable sample size that showed epidemiological information from almost 75% of all live births during 10 years in RS state. The other 25% of newborn babies were screened in private outpatient services, and data from this population were not freely available, as Kopacek and cols. already described in a previous study on another neonatal disease screened ( 34 ).

As well as, the database was managed by the main researcher with support from only one additional person trained for this purpose. Still, the study has some limitations inherent to studies with a retrospective design. The data were obtained from medical records, which were recorded by the medical team. The data about ethnicity was insufficient, not allowing the analysis of this variable. Moreover, associations of CH with post-term ( 24 ) and preterm birth ( 13 , 26 , 27 ) were not analyzed because the information about gestational age was collected organized into categories. Some CH screening services have specific protocols for newborns who are premature, twins, or with low or very low birth weight ( 26 , 35 ). The special protocol for this population is under development by experts of the RSNS-RS and will be implemented in the near future.

In conclusion, this study revealed relevant epidemiologic information from the RSNS-RS. The CH incidence (1:2,377) reported in the present study is slightly higher than the one published in 2009 (1:2,818), despite different laboratory methodologies and protocols for neoTSH values. Newborns with neoTSH values ≥ 20 mIU/L deserve faster diagnosis and therapy initiation, while those with levels ≥ 40 mIU/L must initiate hormone replacement therapy as soon as possible, even without confirmatory tests.
